# Movement building responses to COVID-19: lessons from the JASS mobilisation fund

**DOI:** 10.1007/s40888-021-00241-9

**Published:** 2021-08-28

**Authors:** Awino Okech, Shereen Essof, Laura Carlsen

**Affiliations:** 1 SOAS, University of London, London, England; 2 Just Associates (JASS), Washington, DC USA; 3 Just Associates (JASS), Mexico City, Mexico

**Keywords:** Localisation, COVID-19, Feminist movement building, Feminist leadership, Humanitarian responses, I14

## Abstract

This article draws on the work of Just Associates (JASS), a feminist movement support organisation that strengthens the leadership and organising capacity of community-based women networks in Southern Africa, Southeast Asia, and Mesoamerica, to transform the structures that perpetuate inequality and violence. We analyse qualitative interviews and surveys drawn from recipients of the JASS mobilisation fund (JMF), an innovative financial crisis support mechanism for feminist movements. We argue that localisation strategies deployed by women’s networks supported by the JMF in response to COVID-19, challenge dominant humanitarian responses that de-centre feminist movements, local knowledge, and expertise. By accounting for local knowledge generated from long histories of movement building, building collective power, and challenging racialised and gendered responses to humanitarian crises, women’s collectives and networks supported through the JMF developed contextually relevant responses that challenge patriarchal structural barriers heightened by COVID-19.

## Introduction

As COVID-19 spread across the world, governments generally responded with restrictive mitigating measures such as social distancing, curfews and lockdowns often accompanied by heightened policing and securitisation (see Okech, Mwambari & Olonisakin, 2020). For feminist movement builders, COVID-19 like HIV/AIDS was a human security concern accompanied by a rise in domestic violence and increased unpaid labour as a key gendered feature of how women experienced both the pandemic and government responses to it (UN Women, 2020). However, the global measures to mitigate COVID-19 were not designed to deal with the material concerns associated with the impact of the pandemic. Instead, communities had to grapple with the real threat of illness and death posed by this novel virus alongside concerns about income, food, housing, and water. Unlike classical humanitarian disasters such as floods, hurricanes, drought and famine, COVID-19 is a slow and cumulative crisis for which international relief on the scale of other health or natural crises has been slow. JASS partners in non-welfare states have relied on mutual aid programmes to mitigate the material impact of COVID-19 such as loss of income and therefore associated food, housing, and wellbeing concerns. The analysis we offer in this paper illustrates the role of feminist leadership in developing responses to COVID-19 that models an approach to localisation that centres deep contextual knowledge and a complex understanding of power and gender inequality.

JASS has a long history of supporting women’s organising during the HIV/AIDS epidemic, which it draws on in its current support for feminist movements responding to COVID-19. JASS HIV/AIDS work started in 2006 in Malawi resulting in the “Our Bodies Our Lives” campaign in 2012, a movement that aimed at improving access to safer anti-retroviral medication and quality health care for women living with HIV. The campaign was catalysed by evidence of a global AIDS response that had not acknowledged the gender dimensions of the pandemic and was excluding women’s specific needs, thus exacerbating gender inequalities (Essof et al., 2013).

This paper uses racialised disaster patriarchy and collective protection as frameworks that underpin our analysis of feminist interventions to contain the spread of COVID-19 in sections of South Africa and Zimbabwe. Racialised disaster patriarchy (see Luft, 2016) as a framework facilitates an understanding of the political economy of disasters while collective protection is a framework that responds to the underpinning logics of structural inequalities. There are two major arguments we make in this article. The first is that feminist movement-building strategies are critical to sustaining communities when they encounter humanitarian crises, such as what we are witnessing with COVID-19, due to a history of responding to structural inequalities and deep contextual knowledge. The second argument we offer is that feminist movements exercise leadership by centring collective protection as a framework to mitigate risk, empower, sustain, and strengthen communities dealing with the long-term impact of structural inequalities exacerbated by COVID-19. Collective protection refers to interlinked autonomous responses that draw on context specific indigenous knowledge systems that counter state and non-state attacks on women's rights activists (see Miller et al., 2006: 5). Consequently, these strategies are always invested in understanding and destabilising dominant power structures. Collective protection offers an expanded vision of human security.

We develop these arguments across three sections in this article. In the first section we set up the conceptual framing through a review of critical literature on gender and humanitarian responses, racialised disaster patriarchy and collective protection. In the second section, we examine the JASS Mobilisation Fund (JMF) set up to support feminist community-based formations and movements during crisis. In the third section, we turn to empirical illustrations of the strategies to mitigate the impact and spread of COVID-19. Organised around two primary themes: we examine strategies around material concerns such as food, medicine and protective equipment, and violence against women. Finally, we offer concluding reflections on the lessons we can learn from feminist movement building approaches to deal with health crises.

## Conceptual contours

### Localisation in humanitarian responses

Dominant literature on the humanitarian sector points to a consensus on the gaps between practice and outcomes, particularly the absence of local actors also known as localisation (see El Taraboulsi et al., 2016; Emmens & Clayton, 2017). At the centre of localisation is the need to bring local efforts and responders to the fore of humanitarian action. Local actors are often first on the scene as responders and understand the context better (Roepstorff, 2020: 284). The Humanitarian Policy Group notes that the value of locally led responses lies in access to and deeper networks, coupled with ‘understanding the history, cultural and geopolitics of the situation’ (Humanitarian Policy Group, Overseas Development Institute and International Council of Voluntary Agencies, 2016: 3). There are three major obstacles encountered when localisation is not done well. The first obstacle is connected to limited knowledge of affected contexts by international organisations. As Roepstorff (2020) maintains, there is an inadequate understanding of cultural sensitivities or specific vulnerabilities in the responses by humanitarian organisations. The second obstacle concerns financial aid, with local organisations often not receiving equal amounts of funding. Whatever funding is received is often tied to donor requirements, which tends to ignore what is needed while reshaping the agenda of local organisations. Many local organisations do not receive the bulk of international funding because they do not have the administrative infrastructure to receive and manage finances because they do not operate on a scale that international organisations can adapt to while meeting cost–benefit goals. This long-standing dynamic associated with donor funding creates an unhealthy tension between accessing funding and carrying out the work they want to do. The third concern lies in a poor understanding of the link between gendered needs and underlying gender inequalities.

### Gender and localisation

Efforts to recognise the role of the local through the “grand bargain” which is understood as an attempt to improve effectiveness and efficiency of humanitarian action by centring local actors have excluded women and women’s organisations as responders (Lafreniere et al., 2019: 190). Gender mainstreaming in the humanitarian sector continues to be inconsistent, often lacking a robust power analysis that accounts for gender, age, disability, ethnicity, and other intersecting factors (Lafreniere et al., 2019: 188). An inadequate intersectional response by humanitarian actors is evident in the imbalance between addressing women’s issues and priorities versus donor requirements. The practice and response to disease outbreak has been disproportionately gender blind. Smith points to the material and social impact that gender dimensions of disease outbreaks have (see Smith, 2019). For instance, the lack of investment in accessible public health systems disproportionately skews care responsibilities towards women. Al-Abdeh and Patel (2019: 244) build on this argument by pointing to the challenges that emerge due to a lack of understanding of women’s rights-based approaches to programme planning and delivery. Women are framed only as victims and not as active agents, which is typically seen in the insistence on capacity building thus excluding those who are affected from playing a role in ‘reshaping their worlds’ (Al-Abdeh & Patel, 2019: 248).

Luft advances this view noting that the absence of anti-patriarchal practices during disasters leads to the advancement of racialized gender injustices during the disaster and disaster recovery (Luft, 2016: 7). Luft continues that while racialised patriarchy undergirds the society, disasters unleash, concentrate and justify patriarchy more prominently (Luft, 2016: 7). Reflecting on post-Katrina in the United States of America, gender binaries and inequality within social movements skewed focus towards Black male leaders who led bigger and better resourced movements. The focus on Black male led groups resulted in the absence of gender analysis and feminist practices thus creating an obstacle to intersectional organising. Consequently, the political vision and practice of local women and feminist organisers was left out. ‘Racialized disaster patriarchy’ as developed by Luft focusses on understanding how ‘gendered patterns produce disaster’ (Luft, 2016: 8). Racialized disaster patriarchy centres the intersection of gender (sexism and feminism) and race (racism and anti-racism) as exposing the factors responsible for structural and cultural barriers to post disaster feminist and racial justice work (Luft, 2016: 7).

Racialized disaster patriarchy is a framework that is attentive to the ‘political, institutional, organisational and cultural practices that converge before, during and after disaster that produce intersectional gender injustice’ (Luft, 2016: 7). Racialised disaster patriarchy facilitates an examination of how disasters in addition to exacerbating existing inequalities stack the odds against the intersectional leadership of Black women (see Luft, 2016: 8). Luft (2016) illustrates this phenomenon in New Orleans post-Katrina, noting that a gender-neutral response meant that two years after Katrina, 90% of the Black women leaseholders were dispossessed because public housing was not re-opened. The work to prevent mixed income units was articulated as a race and class cleansing issue obscuring the gendered effects of the disaster (Luft, 2016: 17). Gender-neutral responses of this kind default to centering the experience of men. The barriers that Luft (2016) highlights above which are underpinned by racialised disaster patriarchy are challenged by a model for building collective power for protection as developed by JASS and allies.

Emerging from feminist movement building work in JASS’s three regions, JASS defines collective power as “the ability to create change through community-based organising, alliance building among diverse constituencies and galvanizing networks of allies around shared vision and agendas” (Miller et al., 2006: 14). Further, collective power for protection is deepened by an understanding of and response to increased violence targeted at Women Human Rights Defenders (WHRDs) (see Chavez and Lopez Cruz, 2021; Savage & VeneKlasen, 2018; Penchaszadeh et al., 2013). Savage and VeneKlasen (2018) note that the rise in attacks on WHRDs is the result of rising repressive states, impunity, and religious extremists. Attacks on WHRDs have been captured more recently within discourses on closing civic space, which refers to a series of discrete yet similar patterns of restrictions by governments on civil liberties. These restrictions include but are not limited to physical harassment and intimidation, including threats, injuries and killings, impunity and lack of protection; criminalisation, prosecution and investigation, preventative measures such as terrorism lists and terrorism task forces; administrative restrictions on NGO registration and operation; stigmatisation and negative labelling, including criminal and social stigmatisation of specific actors and ‘space under pressure’, including through co-optation and the closure of newly created space (See Buyse, 2018). JASS and partners propose three major pillars to collective protection as a framework that responds to structural violence (see Lopez & Bradley, 2017). First is the creation of networks which serve as a space for information sharing and strategy development (see Lopez & Bradley, 2017). Second is sustained movement leadership and training as vital to the protection and safety of women in collectives (Lopez & Bradley, 2017). Third, is the creation of safe spaces for solidarity and support, where WHRDs can be in community, share their fears, risks and tactics, while being sustained by friends and trusted allies (see Barcia, 2014; Lopez & Bradley, 2017).

The JMF as a mechanism and the responses supported by JMF, which we focus in this article, respond directly to the three obstacles to localisation noted above by centring local actors’, placing money in the hands of local actors, quickly and without conditions. We also illustrate how an understanding of the third obstacle, which is associated with the instrumentalization of gender is undergirded by the political economy of disasters via racialised disaster patriarchy. Collective power and protection as a framework and strategies therein are a direct response to the architecture of racialised disaster patriarchy illustrating how a feminist response challenges and redistributes power.

## The JASS mobilisation fund: supporting responses to COVID 19

The JMF was established in 2019 as a crisis response fund to provide small-scale, immediate support to ensure the sustainability of women’s organizing responses to food security, health, rights and safety for all. As part of JASS’s feminist movement-building model, the JMF provides emergency funding as one element among a range of resources that include: linking to other sources for longer-term financial support if needed, solidarity, connections for building strategic alliances, tools and strategies for personal and collective care, with the goal of building collective power and protection in times of crisis. The JMF is rooted in the understanding that women are often on the frontlines of community response efforts, playing a critical role in addressing immediate community needs whilst advancing their organizing agendas at the same time (see Ferreira, 2014; Essof et al., 2013).

Although the JMF pre-dated the COVID-19 pandemic and JASS is not a funder, its vision of providing agile, micro level support at critical moments for ongoing organising forms part of how JASS understands feminist movement-building support. JASS activated the fund to support women’s responses to COVID-19, drawing in large part on previous experience of supporting women’s movements during the HIV/AIDS epidemic. HIV + women’s organising revealed that related human security concerns within most groups of people living with HIV and AIDS, went beyond the epidemic itself and included lack of access to fertilizer, land, credit and good healthcare and challenges of stigma and violence which were hardly captured in dominant programme interventions (Essof et al., 2013). These obstacles were exacerbated by inequality, undergirded by discriminatory norms and institutions, which included rigid, traditional gender roles and taboos around sexuality. The function of powerful institutions and people in shaping access to information, education and resources was central to how women experienced living with HIV/AIDS and key to reconfiguring their powerlessness in relation to structures that heightened their insecurity.

JASS work in Malawi through the “Our Bodies Our Lives” movement led to three major conclusions pertinent to the COVID-19 pandemic. The first is that women’s leadership is key to confronting crises at both the macro and micro community levels. Secondly, building collective power from a holistic human security approach is the most effective way to understand and confront a public health crisis. As JASS observed in Malawi, education and organising strategies that enable women discover and strengthen their own resources are essential, along with leadership skills and information to analyse and develop effective strategies to mitigate risk and ensure safety (Essof et al., 2013). Third, long-term feminist movement building strategies are more effective because they contribute to immediate relief and long-term sustainability of communities by challenging inequalities.

The JMF as a movement support response delivers relatively small amounts in cash immediately. The importance of the JMF approach is critical in a global context in which surveillance of foreign aid  including freezing civil society organisations bank accounts  as part of a broader effort to curtail civil liberties challenge at best, but at worst severely limit how financial resources flow (see Chowdhury & Rosencranz, 2021). Through a combination of local networks, innovation through formal and informal remittances JASS gets money to recipients when larger donors have not been successful. The fund disburses between US$500 and US$3000 within 24 h—a time frame generally unheard of among donors and often critical to response in moments of crisis. Many grassroots organizations do not have the formal registration, staff capacity or administrative infrastructure that large donors require to access their funding. In addition, most mainstream funding organisations  take weeks or months to process funding requests, thus prohibiting a timely response to crisis for activists and organizations.

The JMF forms part of broader interventions by feminist funds to shift the skewed power relationships that shape relationships between donors in the global North and partners in the majority world (see Bosch & Bofu-Tawamba, 2019; Clark et al., 2006; Dolker, 2021; Miller & Jones, 2019). A central part of shifting the power in the funding ecosystem  is the emphasis on long-term strategic funding to support feminist movements and organizations based on the recognition that structural change cannot be achieved through short term projects (see DFID, 2018). Despite the discursive shifts, feminist and women’s rights work remains underfunded especially in the global South in comparison to that of other NGOs. An Organisation for Economic Cooperation and Development (OECD) study noted that “in 2016–2017, the 30 members of the OECD Development Assistance Committee (DAC) committed an average of USD 44.8 billion per year focused on gender equality and women’s empowerment as either a significant or principal objective” (Organisation for Economic Cooperation and Development, 2019: 1). Funding for programmes with gender equality as the main objective accounted for only four percent of the total funding (Organisation for Economic Cooperation and Development, 2019: 1). Despite new funding commitments made, women’s rights organizations receive only 0.13% of the total Official Development Assistance and 0.4% of all gender-related aid (see Dolker, 2021). By providing crisis-based solidarity funding, the JMF supports immediate needs as defined by movements, rather than long-term ongoing work. These funds ensure safety and strengthen women’s leadership, which the JMF considers crucial as a response mechanism to COVID-19.

There are four basic criteria that guide the JMF:

1. Recipients must be local groups or individuals located in the contexts in which JASS has established feminist movement-building work and organising who have limited access to funding.
2. Funds must support actions defined by those most affected.
3. Funds should support timely actions/needs and should not be directed toward long-term or ongoing program work.
4. Funds must be disbursed in a way that does not endanger our collective movement-building work for long-term solutions.

Within these criteria, the JMF originally defined eight types of support (1) Activists facing arrest or imprisonment/threats from state/other actors, (2) families of ally activists upon death, imprisonment and other emergencies, (3) wellbeing and health emergencies of activist allies, (4) relocation, legal support and safety plans for activists facing threats, (5) emerging needs, such as shelter and food, following natural disasters such as flooding and fires or community displacement due to extractive projects, (6) organising efforts through transport, communications, stipends (7) local advocacy actions by allied activists/community organizations to elevate demands or raise more money, (8) activists sharing their experiences and leadership on media platforms. A ninth area of support specific to COVID-19 was added to support community-based women and their organising responses to COVID-19 as critical first responders.

The financial support as is evidenced above does not function in isolation, but as a catalyst for a web of strategies and actions by movements and other actors who fund the longer-term work. This rationale is based on the recognition that there are critical moments that can make or break women’s movements and assure the sustainability of feminist work. The criteria and areas of support reflect the fundamental principles of JASS’s feminist organizing with women in movements. There are three main ones that we would like to foreground. First, the recipients are all based in the regions where JASS has worked for more than a decade and have an existing relationship with JASS. This pre-existing relationship ranges from participation in JASS feminist leadership schools to ongoing partnerships in movement strategy support, campaigns, and actions from local to global levels. Second, that there is a rapid delivery of funds in cases where organizations do not have access to other financial resources. Funding decisions are made rapidly due to JASS existing relationship with local movements. Third, the system of categorizing grants highlights the role of the JMF in tracking and identifying specific challenges arising in feminist organizing across the regions JASS works in. The JMF creates real-time knowledge of the contexts and the impact of events on women’s organized responses. This knowledge aids feminist organizing not only in moments of crisis, but also over the long term.

As shown in the Table 1 below from April 1 to December 30, 2020, a total of US$174,677.00 was disbursed to 116 recipients from the JMF. There were 59 recipients in the Mesoamerica region (Guatemala, Honduras, Nicaragua and Mexico), 34 recipients in the Southeast Asia region (Philippines, Cambodia, Thailand, Indonesia and Myanmar) and 23 recipients in the Southern African region (Zimbabwe, South Africa and Malawi).

**Table 1:** Money disbursed between April–December 2020

Region	Country	Number of groups/individuals	Total disbursement/USD	Recipients
Mesoamerica	Guatemala	22	42,873	59
Mesoamerica	Honduras	22	19,725	
Mesoamerica	Nicaragua	4	9000	
Mesoamerica	México	11	16,200	
Southeast Asia	Philippines	20	24,245	34
Southeast Asia	Cambodia	5	8700	
Southeast Asia	Thailand	1	1000	
Southeast Asia	Indonesia	6	11,064	
Southeast Asia	Myanmar	2	3000	
Southern African	Zimbabwe	5	11,500	23
Southern Africa	South Africa	13	18,000	
Southern Africa	Malawi	5	9370	
			$174,677.00	116

Four months after disbursing the money, JASS conducts surveys with recipients of the JMF. In this case a survey was conducted with 37 partners between August–September 2020. The surveys focused on how communities were doing, the shifts in power, knowledge built, and networks strengthened. The questions included: what were the funds used for and what changed as a result? What else did JASS support; and what did you learn? JASS also conducted follow up conversations in May 2021, which were both an expression of solidarity and an opportunity to assess the impact of the emergency funding on long-term movement-building work. In the follow up conversations, JASS focussed on the significance of the JMF solidarity money; how the choices made with the money shifted the community's experience of COVID-19 What aspects of the experience of COVID-19 were mitigated by their interventions; how did the work done during this period strengthen their long term strategies for social change and what has the COVID 19 pandemic taught them about their approaches/strategies and the socio-economic and political environment they operate in.

Our analysis in this paper is informed by a thematic analysis of the qualitative narratives from the proposals, surveys and follow up conversations. In reflecting on this qualitative data, JASS like other feminist organizations draws on a long scholarly and movement-based critique of outcome-based understanding of structural change that relies on linear before and after narratives of change to measure impact (see Brisolara et al., 2014; Sielbeck-Bowen et al., 2002; Wakefield & Koeppern, 2017). JASS understands the structural issues that movements respond to as complex, deep rooted and transforming these conditions requires multi-faceted, networked actions. Attribution is not a simple task and the JMF is designed to circumvent the expectation that recipients should offer evidence of how small amounts of money has changed their lives. Instead, we place resources in their hands to meet the needs that they identify as critical to sustaining their work and lives. This article therefore approaches an engagement with impact through a recognition that the injection of much needed financial resources in times of crisis to serve under resourced and marginalized communities arrests a situation that would have escalated negatively. By examining how racialized disaster patriarchy (see Luft, 2016) is challenged by local actor’s crisis responses through collective power and protection, we illustrate the importance of innovative local feminist leadership movement building approaches.

## Strengthening medical and food supply

“At first, the COVID was just an unpleasant rumour. It became a reality when we saw posters on COVID shared in all the community spaces […] and then, the lockdown came. It was awful because vending was not allowed, and bars and beer halls were closed, schools were closed, and in our area, the local clinic was also closed because the healthcare workers were not prepared to deal with the new virus on their own without support from Government. They had to go and protect themselves and their families too. Our local police post was also closed, and we were forgotten. Violence increased; it was crazy as cases of violent crimes rose. We did not have money because our vending stalls were closed, and even if they had not closed, the people did not have money. Hopely is an informal settlement, and most of the people here work in the informal sector.” - Sex worker organiser, Hopely, Zimbabwe, May 2021

“We don’t know how many people died from cholera, typhoid and other disease outbreaks because we do not have proper health facilities here. We only have one clinic which serves the whole Hopely Settlement, and it is often overcrowded. The overcrowding on its own is a threat to the containment of COVID-19. It is difficult to do physical distancing even at the clinic because there are too many people who need assistance and too few health workers. We do not even know how many people have COVID-19 because there is no testing facility here" - Sex worker organiser, Hopely in Zimbabwe, January 2021

The excerpts above from sex worker organisers in Hopely Zimbabwe point to the impact of a socio-economic and political crisis that begun in the 1990s and escalated through the withdrawal of multilateral funding to the country by the Bretton–Wood institutions, a succession of fractured bilateral relationships with Western governments and shrinking civil liberties (see Besada & Nicky, 2008: 2–3). Subsequent hyperinflation led to a rise in the cost of basic goods and services, unemployment, and a slow disintegration of public infrastructure—health, education, housing among others. In addition, the economic strategy of “looking East” because of the fracture in relations with Western governments, opened the country to predatory natural resource extraction in exchange for economic investments (see Besada & Nicky, 2008). The impact of high rates of unemployment, high cost of living and predatory extractivist policies, accompanied by large-scale land grabs reverberates across spatial divides in the form of displacement within Zimbabwe, a large diasporic global community and generalised economic insecurity (see Besada & Nicky, 2008; Win, 2013: 22). Successive Zimbabwean governments have disenfranchised citizens through greater controls on freedoms associated with political organising and expression of dissent (see Chitanana, 2020). Concomitantly, there is also a reprisal of gendered discourses to reframe the “soul” of the nation. As Mupotsa and Lennon (2008: 99–100) note, the construction of conservative patriarchal notions of Zimbabwean womanhood that rely on “morality” and “respectability” are mapped onto the “nation building” project. Consequently, policies are developed to target sex workers and “non-respectable” women in urban areas ostensibly to “clean up cities”. All the while multiple sexual partners for men is  viewed as acceptable.

JASS solidarity in Hopely specifically and other parts of Zimbabwe is rooted in a feminist analysis of how gender is mobilised for nation and state building. Focusing on sex workers, women in urban informal settlements, cross border traders and women living with HIV/AIDS, JASS is invested in strengthening activist leadership, supporting building of community networks and bolstering partner organisations in formulating innovative approaches to economic livelihoods. JASS allies and partners organised to mitigate the impact of COVID-19 given the prevailing socio-economic and political conditions described above. JASS support to the collective of sex workers in Hopely over the last 5 years through study circles, feminist movement-building schools and tactical dialogues has facilitated their ability to organise around better housing, legal, safety and health services as is evidenced below. In essence, the ability to mobilise quickly around a COVID-19 response was enabled by a longer history of organising and community building by the group. In addition, the Hopely experience points to the interconnected crisis that COVID-19 accelerated in informal settlements such as Hopely. The escalation of a health crisis connected to pre-existing terminal health conditions meant that lockdown measures that cut off income and food supply would only accelerate non-COVID-19 related deaths.

“When we got the money, there were very pressing issues amongst us as a group that needed urgent attention. We needed to have access to Anti-Retroviral Therapy (ART), or we would die. We had a group that focused on taking turns to go and get ART for everyone. We came up with a WhatsApp group to quickly share information when we were facing violence or any threats. We bought seed and seedlings, and we planted vegetables, garlic and other plants in any container that we could get so that we can be able to have something to eat. We used the money to generate more income, we had a mask-making project where we made washable masks, and we had a stokvel (money-go-round) so that we could lend each other money for income-generating projects at an interest of 20%. The profit we received is what we used when one of us had to go to collect our medications. We dug a community well with some of the money so that we have water that we can use for drinking and other household use—the well-provides water to water our plants as well. Instead of buying sanitizers, we bought soap, and this assisted us to make sure that we maintain the clean homes and communities as required by the community healthcare workers.”- Sex worker organiser, Hopely, Zimbabwe – January 2020

The interventions prioritised by Hopely sex workers are linked to systemic inequalities that are exacerbated by COVID-19 mitigating measures that are inattentive to the income disparities in the country. Surviving COVID-19 in this instance is not framed as a conversation about the pandemic but primarily about staying alive despite COVID-19. Consequently, we see the impact of flexible solidarity funds such as those provided by JMF in enabling local actors to make decisions around what is needed by communities in that moment, rather than a COVID-19 fund that may have prescribed that resources are used for masks and sanitisers.

Similarly, in South Africa, the JMF support in Ocean View went towards women’s organising in informal settlements to curb the spread of COVID-19, income generating activities as well as support for the overall wellbeing of the community. The Ocean View settlement was established 70 km from the Cape Town city centre by the apartheid regime in the late 1960s, under the Group Areas Act of 1950 that legislated race-specific townships (Pinnock, 1989: 157). The women of Oceanview rezoned the settlement to facilitate community management of the pandemic. They established a green zone on the far end of the settlement for those most vulnerable to COVID-19, a yellow zone as a buffer to house those who were less vulnerable, and a red zone for those who had contracted COVID-19 and needed care. They also established a safety committee that opened a soup kitchen to provide food for impoverished families in the settlement.

We have 54 houses in this informal settlement. The mothers against gang violence met and made agreement with these people. We have many people who are siek en swak (sick and weak) and need to be protected from Aunty Rona. We made four spaces separate for these people. Then those who are strong they must stay next, then those who have children and who are not moving out they must stay next, those who are siek and swak not from the rona they must stay together. They say zones so we also said okay th[ese] will be zones, red for those sick by the rona, then yellow for those we must protect and then green for those who are strong. – Organiser, Mothers against Gang Violence, Oceanview, January 2020

We made a truce with the gangs in lockdown. As mothers we said no, we cannot live with the rona and the gangs together. We met with those skollies [gang members], we know them and we said in this area the wars and the shootings have to stop while Aunty Rona is visiting. A truce. We could expand our community garden also. We grow many things there that we can use in the soup kitchen, especially carrots and cabbage and potatoes and those herbs that the rasta sell to keep healthy. - Organiser, Mothers against Gang Violence, Oceanview, January 2020

The strategic actions of the women in Oceanview, reflect organic tactical public health responses through a rezoning procedure which accounts for overcrowding, poor sanitation, and the absence of effective state interventions to respond to those on the margins of the society. While these interventions were not set up to systematically track COVID-19 infections and containment, the strategy worked to make the management of infections easier and reduce risk across the settlement as evidenced below. In addition, the door-to-door services were responsive to the impossibility of social distancing in overcrowded household environments. The combined action of zoning and door to door services resulted in reduced infection rates.

Three people got the rona and we could move them away from the others and look after them with distance. If we didn’t do this everyone in this area will get sick. This was a good thing we did to keep safe and care for others. No one died. We also made masks. One of our members used to work there by the clothing factory in Salt River. She has a machine so we bought materials with some of the money and made masks, we would sell them for 5 rand and then also give away to people. So we made sure everyone had a mask to wear to protect. No one could come in here without wearing a mask. - Organiser, Mothers against Gang Violence, Oceanview, June 2021

The families inclusive of elderly, frail, disabled and children of our "Uber Feets" visited, besides some having chronic illnesses due to the zoning and diligent work of the "Uber Feets", their health status never changed for the worse. The daily door- to-door service provided had families being more hygienic in an overcrowded restricted space at home. There would have been many violations of the restrictions on movement during lockdown. An increase of possible contamination and more frequent visits to Health Facilities that are expensive as we do not have them here and anyway they were already overburdened*.* - Organiser, Mothers against Gang Violence, Oceanview, June 2021

Women organisers in Oceanview reveal an attentiveness to how power circulates in society through interventions that focus on how the most marginalised experience insecurity from the institutions that are ostensibly set up to secure them. The long history of organising by *Mothers against Gang Violence* gives them the gravitas to negotiate with the community around zoning despite the inconvenience. The women also facilitate a truce with gangs. Like Hopely, the systemic inequalities that are unmasked by COVID-19 are visible in the tactical approaches adopted by the women who respond to material realities such as lack of income and therefore food before addressing the risk of COVID-19. The interventions from Oceanview and Hopely draw clear links to the structural violence experienced by these communities, which is seen in the long-term impact of privatisation and subsequent divestment by governments in public services leading to the destruction of health, housing, water and other basic needs. To assume that the largest crisis for people in Oceanview and Hopely was COVID-19 is to ignore the fact that the pandemic only heightened existing interconnected crises that successive local and national governments had failed to effectively respond to. The responses by the women organisers centre gender by collectively organising at a strategic and basic needs level.

This intersectional response as evidenced in the excerpts above, challenges racialised disaster patriarchy (see Luft, 2016) because the women are attentive to how gender, race and class work together to heighten vulnerability during disasters and in this case a public health pandemic. It is at the nexus of gendered and racialised marginalisation, that the collective and locally rooted responses emerge. The mothers in Oceanview and the organisers in Hopely are responding to existing vulnerabilities generated by class on the one hand—leading to income related and survival interventions around material concerns such as food—and gender, which is evident in the care responsibilities assumed  by the organisers to build collective safety and security in the settlements. The unpaid care work seen in these organisers community interventions is a feminist leadership intervention during crisis because community mobilisation sits at the centre of their actions.

The feminist movements JASS works with tapped into the reservoir of justice and freedom work to serve as first responders with the solidarity resources provided by JMF producing localisation that was based on contextually relevant interventions that not only mitigate the impact of COVID-19 for women as a group but also extend to the broader community. Feminist organisers in Oceanview as shown below draw on a complex understanding of power and collective protection to disrupt racialised disaster patriarchy by providing leadership through holistic community protection strategies.

“Due to the restrictions, we used the concept of Home-based Care/ Meals on Wheels to provide a service to the settlement that was beneficial to the residents. The greatest concern for us was the gangs and to keep our “Uber Feets” healthy and happy even though it was done voluntarily. If it wasn’t for Uber Feets, there would have been congestion of lines of people which would have been a major problem with the restrictions stipulated by government and enforced by the police, who was harassing people in our communities. So the Uber Feets actually was the best thing ever to have been born” - Organiser, Mothers against Gang Violence, Oceanview, June 2021

We had daily Health Sanitation Awareness with families of “Uber Feets” and today people are still maintaining it. Our kitchen is still going. Our work with the children is still going and our tensions with the gangs are still going. Today because of intervention we have people that attended rehab facilities and they still have a clean bill of health. We have families that started their own vegetable gardens, arts and crafters, etc. - Organiser, Mothers against Gang Violence, Oceanview, June 2021

The localisation model illustrated above underscores how ignoring the gendered impacts of any crisis reproduces inequalities rather than redistributing power. The redistribution of power is evident in the collective protection strategies, which through a combination of autonomous actions and strategies respond to structural violence, draw on deep knowledge of the communities and leadership within them. Finally, the longevity of these responses as seen in drug rehabilitation, food gardens, sanitation strategies is grounded in the organic emergence of actions rather than a prescribed programme formulated by external actors.

## Confronting violence against women

The second major area that evidences collective protection as a direct challenge to racialised disaster patriarchy are interventions dealing with the rise and threat of violence against women during the pandemic. While addressing the immediate problem of increased violence against women, the focus in the interventions we examine below remained on the broader socio-political context that existed before COVID-19, which rendered responses by the state to these specific issues unreliable, weak and a risk for women. The organizations examined below developed strategies around gender-based violence and COVID-19 management in contexts shaped by various forms of regime surveillance through militarised enforcement of curfews and pre-existing gender based socio-economic exclusion. JMF supported interventions focused on mitigating gender-based violence in South Africa, were based on the recognition that the lockdown policy would compound the high levels of generalised violence against women. In South Africa, one woman is murdered every 3 h, which is five times the global average (SAPS, 2019). Official figures underreport the full extent of the violence, especially during lockdown (Amnesty International, 2021). In Gauteng and Limpopo, Rise up against gender-based violence collective emerged from the total shut down movement, which was a national mobilisation demanding the government's commitment  to ending violence against women (UN Women, 2018). Total Shut Down called for the collective withdrawal of women’s labour from the economy until the government took stronger and sustained measures to address gender-based violence (UN Women, 2018).

Rise up observed that with COVID-19 lockdown measures women were trapped in homes with their abusers without access to immediate support of friends and family (see Rehse & Mchuchu-MacMillan, 2020).

“JASS was among six to seven organizations that released emergency funds to us. Rise Up does three things: emergency evacuations of GBV survivors, behavioural change programmes, and provide direct support to women living on the streets. JASS specifically supported us in two things – address issues of women living on the streets and evacuate some survivors. We don't own a vehicle, so a rental vehicle was required for evacuations (actually going physically and remove survivors from their homes). - Rise Up organiser, May 2021

Given lockdown conditions, Rise Up secured essential service permits so that they could run the evacuation service for survivors described above. As evidenced in the excerpt above and below, the organisers undertook work that determined life and death conditions for victims of violence. In addition, they delivered food packs, cleaning products, and sanitary products to victims of violence who were trapped with abusers who were withholding money or food as a form of punishment.

“To be honest, we do a lot of life and death work. One woman died, unfortunately; we did lose one of our survivors. She had called to say she didn't want an evacuation. She just wanted us to make sure that the police got to her on time because she was in a physical altercation with a partner. And she said that she feels like she's going to kill him. By the time the police had gotten there, he had killed her. Another one of our survivors was seven months pregnant and had been held hostage by her abuser since she was two months pregnant. One day when he had to leave the house, she managed to access her phone and let us know that she needed help. We went to South African Police Services to support us in evacuating her. Both her and the baby (now eight months old are fine) in a shelter and the abuser has never been able to find them because of the method that we use to make sure that they're not able to track our survivors. In such cases, we're quite grateful of that kind of impact of literally saving, we saved a baby's life. " - Rise Up organiser, May 2021.

“We prevented GBV deaths. Our long-term goal is to make sure that women don't die at the hands of men. That's what the fund helped us to contribute towards. I also think it helped us to identify gaps in the system, for example, like the issue around the WhatsApp line and the GBV Command Centre. That's what has created some level of sustainable change because there are going to be women whose lives are going to be saved years from now, because they were able to send a WhatsApp instead of making a phone call”. - Rise Up organiser, May 2021.

In Gauteng, the focus of the Precarious Women Workers was also on supporting women facing gender-based violence at work and in the home. The group grew out of a workplace struggle against oppressive labour conditions and sexual harassment at Heineken's Sedibeng brewery. As a result of labour broking, workers at the brewery were employed as temporary casual workers, which meant they were subjected to menial wages with no benefits and given their temporary status were prevented from unionizing (Smit, 2017). Women workers fought against labour broking and exposed a “sex for shifts” system at the brewery where supervisors expected sex in order to be scheduled on good shifts, get a better job, or keep their jobs (Mbude, 2019). The Precarious Women Workers’ COVID-19 response focused on strengthening communication as a safety strategy by purchasing cell phone airtime. Through weekly WhatsApp meetings, the group sought legal support as they prepared a risk assessment and analysis to inform their organising strategy, which includes creation of collective protection networks and direct action against violence.

“Extra costs were being incurred for communications to support connectedness and physical and mental health, as well as navigating the police who are focused on enforcing lockdown and telling women at risk that they do not have time to get involved in 'family matters'. - Precarious Women Workers organiser, 5 January 2020.

“One of the things that we do are called “go togethers”. Where if a woman is facing violence or harassment from a man and wants to apply for a protection order, which was impossible during the lockdown instead of a woman going alone to the magistrate court or police, we go together with a group of women which allows us all to learn about the procedures and how you need to and can assert yourself. We are also learning where these processes are unhelpful, where we need other ways to deal with GBV. I think this was the biggest shift we made – we already were engaging that but the lockdown and COVID made it so much more urgent because there was this explosion of violence and therefore everybody was aware – it made a lot of sense.” - Precarious Women Workers organiser, June 2021.

The group also conducted self-defence training to support women members in situations of violence.

“I don’t know any other groups is focusing on self-defence. In South Africa where GBV is madness – where women are abused and attacked all the time, it is necessary to build that line of defence. It’s actually surprising how the little attention [is] given to physical safety. I don’t know many groups like us women’s groups focusing on this. PWW came to be because there are institutionalised and common ways of doing things for example when you have a problem at the workplace, you form a union and collectively address these problems but when it comes to GBV, it doesn’t seem to work like that. - Precarious Women Workers organiser, May 2021.

Collectively, these examples from South Africa illustrate how pandemics exacerbate gender violence and discrimination that governments have systematically failed to address, which feminist movements have historically organised around. In South Africa, where the government’s response to COVID-19 failed to account for the gendered socio-economic impact, the work of securing communities fell on feminist movements and organisers. These interventions illustrate the opportunity for holistic responses when we treat gender as a site of analysis that frames strategic responses thereby exposing the limitations of humanitarian responses that reproduce racialised disaster patriarchy (see Luft, 2016). In effect an analysis of how power is mobilised and organised through gender, leads feminist organisers above to develop multi-nodal responses that focus on immediate rescue and survival through material, legal and defence strategies. The excerpts above also point to the importance of building collective power as the basis for the collective protection. Collective power as an approach is rooted in an integrated response strategy that centres safeguarding as a mechanism to decrease risk and vulnerability by shifting focus away from individuals and instead emphasizing group safety and wellbeing.

A central critique of humanitarian responses as pointed out earlier in this paper is that governments and international aid responses short circuit local community strategies. While the importance of the local is emphasized, women are often ignored or their participation instrumentalised leading to greater vulnerability for women in communities that are already impacted by structural inequalities. When interventions are guided by movements and organisations that have deep knowledge of their communities, we see a pattern of grassroots mobilising that can be described as localisation in action. The efficacy of these localisation strategies lies in the injection of financial resources at the right time to groups on the margins of development funding, and governments responses, which leads to measures that were vital to survival.

## Conclusions

We conclude with two main lessons from the JMF. The first lesson is on the importance of solidarity funds such as the JMF to supporting strategic, material, and timely feminist organising during crisis. Movements supported through the JMF note its importance in the excerpts below.

“The significance for this group was that it was a gift of money. I am phrasing in this way because that is something different to a relationship of funding. What that meant is that we received the money very quickly, we didn’t have to look for a fiscal host or register the organization or group – we basically had to give one bank account. Besides that it was quick, what it really meant for us was that there was a relationship of trust which is very valuable”. - Precarious Women Workers, Gauteng, South Africa, June 2021

“Operating during COVID-19 lockdown in an informal settlement with over 500 people was the most challenging, to cope we were improvising constantly. The significance of being a recipient of this funding was that it came at a time when there was major confusion at government and business partner levels. If we didn’t have the money, we would have had a major health crisis given that our health facilities in the country were already overburdened”. - Mothers against Gang Violence, Oceanview Settlement, Cape Town, South Africa - June 2021

Localisation is about centring local actors as knowers in crisis response. The cases examined in the paper illustrate how offering flexible resources and leaving decision making to local actors leads to responses that focus on the needs of the community generally and women specifically. The agency associated with flexible resources means that decisions can be made quickly by local actors to support the most urgent needs as identified by them  and not as determined by externals. The JMF as a crisis response and movement building support strategy should become the norm in humanitarian crisis response strategies rather than the preserve of feminist funds and movement support organisations like JASS.

The second and final lesson as shown in this article is that localisation approaches that centre feminist movements lead to actions that are informed by a robust gender power analysis thus reducing the risks associated with gender-neutral approaches that ignore gendered impacts of crisis. In addition, the evidence offered in this paper also illustrates the value of deep contextual knowledge and a history of organising as facilitating quick and well thought through strategies for communities. The result as illustrated across this paper are collective protection strategies that mitigate the life and death implications of crisis including those generated by pandemics for women and communities on the margins of society. Feminist movements privilege community needs and account for the sources of vulnerability for specific groups. By centring collective power and protection as a set of autonomous integrated strategies for safety, security, and wellbeing, JASS and allies offer a framework that resolves the essentialist integration of women that has characterised mainstream humanitarian responses (see Al-Abdeh & Patel, 2019; Smith, 2019). Collective power and protection as a strategic response framework deployed by the movements the JMF supported, disrupts racialised capitalist patriarchy (Luft, 2016) as the undergirding logic that exacerbates vulnerability and harm during crises.
